# Physical inactivity and anthropometric measures in schoolchildren from Paranavaí, Paraná, Brazil

**DOI:** 10.1016/j.rpped.2014.11.009

**Published:** 2015-03

**Authors:** Flávio Ricardo Guilherme, Carlos Alexandre Molena-Fernandes, Vânia Renata Guilherme, Maria Teresa Martins Fávero, Eliane Josefa Barbosa dos Reis, Wilson Rinaldi

**Affiliations:** a Universidade Estadual de Maringá, Maringá, PR, Brazil; b Universidade Estadual do Paraná, Paranavaí, PR, Brazil

**Keywords:** Sedentary lifestyle, Obesity, Child, Adolescent

## Abstract

**OBJECTIVE:**

To investigate the association between physical inactivity and anthropometric measures in schoolchildren from Paranavaí-Parana, Brazil.

**METHODS:**

Cross-sectional survey, carried out in July and August 2013. Sample of 566 students (287 boys and 279 girls) from 6th to 9th grade, aged 10 to 14 years, from public and private schools of Paranavaí - PR, Southern Brazil. The variables analyzed were: time of weekly physical activity through a questionnaire (physical inactivity <300 minutes/week), body mass index (BMI) and waist circumference (WC). In the statistical analysis, the U Mann-Whitney and Student's *t* tests were used for comparison between genders. To identify factors associated with insufficient levels of physical activity, univariate and multivariate logistic regression analysis was applied and expressed in Odds ratio (OR) and 95% confidence interval (95%CI).

**RESULTS:**

There was an association between physical inactivity and anthropometric measurements for BMI (*p*<0.001) and WC (*p*<0.001), with a prevalence rate of 56.1% and 52.7% of inactive adolescents, respectively. In the multivariate analysis, there was significant association of physical inactivity and overweight (OR 1.8, 95%CI: 1.1-3.0) and with increased waist circumference (OR 2.8, 95%CI: 1.4-3.8).

**CONCLUSIONS:**

Inadequate levels of physical activity is a determining factor for overweight and abdominal adiposity. Accordingly, preventive measures should be taken, especially in schools, emphasizing the importance of exercise for body composition control and weight reduction.

## Introduction

Obesity can be defined, in a simplified form, as a disease characterized by excessive accumulation of body fat, as a consequence of a positive energy balance.^1^ In the last decades, its prevalence has increased worldwide, constituting a major health problem.^2^

Recent data have shown a substantial increase in cases of overweight and obesity in the last 20 years, in childhood and adolescence.^3^ One possible explanation is that biological and behavioral changes occur at this stage of life, among them the adoption of inappropriate eating habits, such as increasing the energy supply through diet and physical inactivity.^4^^,^ 5

Regarding physical inactivity, Brazil shows a concerning picture in the young population, due to the high prevalence found among them.^6^^,^ 7 This concern is exacerbated by evidence that physical activity levels tend to decrease with increasing age, which is known as tracking of physical activity.^6^

Small increases in physical activity are associated with health benefits. In children and adolescents, physical activity can reduce symptoms of depression and stress, improve cardiopulmonary function, muscle fitness and bone health and reduce body fat levels,^8^ with the latter being the main risk factor for the onset of metabolic diseases.

As physical inactivity is usually acquired in childhood and remains in adulthood, its early identification is crucial, as well as its association with anthropometric indicators for better control and prevention of excess weight and its co-morbidities in adulthood. Therefore, this study aimed to investigate the association between physical inactivity and anthropometric measurements in schoolchildren from the municipality of Paranavaí, state of Paraná, Brazil. 

## Method

The population consisted of students from public and private elementary schools of the municipality of Paranavaí, state of Paraná, Brazil. According to data from the Regional Education Center of Paranavaí in 2013, 4,540 students were enrolled in eight public schools and four private schools in town. Paranavaí is located in the northwestern region of the state of Paraná. In 2010, the municipality had 81,590 inhabitants, of which 95.3% lived in urban areas. The municipal performance was classified as medium for the items employment, income, health and agricultural production, and high for the item education. The current HDI is 0.763, and the GDP *per capita* of R$14,180.10. 

This cross-sectional study was carried out in July and August of 2013. The sample consisted of schoolchildren from the 6^th^ to the 9^th^ grades, aged 10 to 14 years, from public (4) and private (2) schools of the municipality. These schools represented 50% of all schools and accounted for 61% of the school population of the municipality. The classes were chosen by systematic random sampling in three steps: 1) selecting one school from each region of the city by drawing lots, to better characterize the school environment of the city, considering that the number of schools and students were similar in all regions; 2) selecting the classes by drawing lots in each school; 3) inviting all students in the selected classes and providing explanations about the study.

The resulting sample was calculated based on the total number of the population analyzed (n=4,540); prevalence of the outcome (physical inactivity) of 50% (unknown); confidence level equal to 95%; and sampling error of 5%. Based on these parameters, it was estimated the necessity to collect data from 354 schools. We added 10% to sampling, predicting losses and refusals, and 10% for the multivariate analyses, resulting in 425 children and adolescents. Evaluations were performed only in those invited schoolchildren who agreed to participate in the study and whose parents/tutors signed the Free and Informed Consent Form, totaling 578 students. Of these, 12 individuals were excluded: 1) age different from 10-14 years; 2) those who did not undergo all assessments. The final sample consisted of 566 children and adolescents, 287 boys and 279 girls. The margin of sampling error, calculated *a posteriori,* was 3.8 to 3.9%, below the value established *a priori* (5%).

The evaluations were carried out during school hours by trained evaluators, using calibrated equipment. Height was measured with a wall stadiometer (WiSO, Brazil) with a resolution of 0.1cm, and body mass in a digital scale (G-Tech, USA) with a maximum capacity of 150kg and a resolution of 100 grams. The subjects wore only the school uniform, without coats or objects in pockets. BMI (kg/m^2^) was used to classify students as adequate weight and excess weight.^9^ Data from children with low weight (0.3%, n=1) were included in the category of adequate weight.

Waist circumference was measured using a flexible non-elastic tape (Gulick, Brazil), with a resolution of 0.1 cm, applied immediately above the iliac crests. For the classification of abdominal obesity (central), we used the cutoff of *p*≥75 for all ethnicities.^10^ It is noteworthy that few studies have used national parameters to classify abdominal obesity in adolescents. As for the level of physical activity, the questionnaire applied by the Brazilian Institute of Geography and Statistics (IBGE) in the "'National Survey of Schoolchildren Health" ("Pesquisa Nacional de Saúde do Escolar") was used.^11^ Physical inactivity was considered according to the cutoff of <300 minutes of moderate/vigorous physical activity per week, according to the guideline of physical activity for adolescents.^12^

For the statistical analysis, the Kolmogorov-Smirnov test was used to identify data normality. To compare the anthropometric characteristics and time of weekly physical activity between the genders, we used the Mann-Whitney U test for independent nonparametric samples and the Student's *t* test for independent parametric samples, accompanied by the Levene test for analysis of homogeneity of variances.

The chi-square test was used to assess the differences in proportion of physical inactivity (dependent variable) according to the categories of the independent variables. Exploratory analysis of data showed non-linear association between x and y, from a certain point of its distribution, assuming a logistic curve in S. Therefore, the univariate and multivariate binary logistic regression was performed, determining the odds ratio (OR) and respective confidence intervals (95%CI), in order to assess the association of physical inactivity (dependent variable) and the independent variables. All studied variables were dichotomized and the criterion for inclusion of independent variables in the multivariate model was an association level of *p*≤0.20 with the dependent variable at the chi-square test.

Analyses were performed using the Statistical Package for Social Science (SPSS), release 20.0, considering a *p*<0.05. This study was approved by the Research Ethics Committee of Universidade Estadual de Maringá, under number 353,552, according to the Declaration of Helsinki. 

## Results

Of the 566 students selected for the study, 50.7% (287) were males and 49.3% (279) were females, with mean (± standard deviation) age of 12.4 (±1.2) and 12.3 (±1.2) years, body mass of 52.0 (±13.8) and 49.5 (±11.7) kg, height 1.59 (±0.1) and 1.56 (±0.1) m, BMI of 20.29 (±3.8) and 20.19 (±4.2), waist circumference of 74.4 (±11.2) and 71.6 (±10.7) cm, and finally, physical activity time of 359 (±401.4) and 343 (±471.2) min, respectively. The boys showed significantly higher mean body mass, height and waist circumference in relation to girls (*p*≤0.05). The mean age, BMI, and physical activity time were similar between the genders (Table 1). 

**Table 1:** Age, anthropometric characteristics and physical activity in schoolchildren from Paranavaí, Paraná in 2013.

Variable	Mean±SD		
	Male (n=287)	Female (n=279)	*p* value
Age (years)	12.4±1.2	12.3±1.2	0.256
Mass (kg)	52.0±13.8	49.5±11.7	0.048
Height (cm)	1.59±0.1	1.56±0.1	0.002
BMI (kg/m^2^)	20.29±3.8	20.19±4.2	0.788
WC (cm)	74.4±11.2	71.6±10.7	0.002
Time P.A. (minute/week)	359±401.4	343±471.2	0.170
PA, physical activity			

PA, physical activity

Table 2 shows that most of the physically inactive individuals were males (53.3%), aged 10 to 12 years (59.4%) and from public schools (67.8%). However, the only independent variables associated with inadequate levels of physical activity (<300 min/week) were BMI (*p*≤0.001) and WC (*p*≤0.001), with prevalence rates of 56.1% and 52.7% of inactive individuals, respectively.

**Table 2:** Sociodemographic and anthropometric characteristics in relation to level of physical activity in schoolchildren from Paranavaí, Paraná in 2013 (n=566).

Variables	Level of Physical Activity			
	n	Total (%)	Insufficiently Active (%)	*p* value
Gender				
Female	279	49.3	46.9	0.323
Male	287	50.7	53.1	
Age				
10-12 years	310	54.8	59.4	0.058
13-14 years	256	45.2	40.6	
Type of school				
Public	381	67.3	67.8	0.839
Private	185	32.7	32.4	
BMI				
Normal weight	339	59.9	43.9	0.001
Excess weight	227	40.1	56.1	
WC				
Adequate	361	63.8	47.3	0.001
Obesity	205	36.2	52.7	

BMI, Body Mass Index; WC, Waist Circumference.

Subsequently, the univariate analysis showed that physical inactivity was positively associated with BMI (OR 3.2, 95% CI: 2.3-4.6) and WC (OR 3.5, 95% CI: 2.5-5.0).

At the multivariate analysis, using logistic regression, it was observed that the model with the highest predictive validity included the variables BMI, WC and age (adjustment with Hosmer and Lemeshow test=0.938), with the capacity to explain 79.5% of cases of students with adequate level of physical activity. The insufficient physical inactivity was once again positively associated with the two anthropometric variables adjusted by age. Students classified as having an increased BMI (excess weight) were 1.8 times (95%CI: 1.8-3.0) more likely to be physically inactive than individuals with normal weight. As for the WC, students with abdominal obesity were 2.2 times (95% CI: 1.4-3.8) more likely to have inadequate levels of physical activity (Table 3).

**Table 3:** Factors associated with insufficient level of physical activity in schoolchildren from Paranavaí, measured by logistic regression.

Variables	Crude Odds ratio (95%CI)	Adjusted Odds ratio (95%CI)
Gender		
Male	1	—
Female	0.9 (0.6-1.2)	—
Age		
10-12 years	1	1
13-14 years	0.7 (0.5-1.0)	0.9 (0.6-1.3)
Type of school		
Public	1	—
Private	1 (0.7-1.4)	—
IMC		
Normal weight	1	1
Excess weight	3.2 (2.3- 4.6)	1.8 (1.1-3.0)
CC		
Adequate	1	1
Obesity	3.5 (2.5-5.0)	2.2 (1.4-3.8)

BMI, Body Mass Index; WC, Waist circumference.

## Discussion

Physical inactivity in children and adolescents has increased around the world, with the highest rates being observed in individuals with excess weight.^6^^,^ 8 Studies have attempted to identify the anthropometric parameter better associated with physical activity levels in children and adolescents, but the results are conflicting.^13^^,^ 14 This study aimed to assess this issue by analyzing the association between physical inactivity and two anthropometric indicators of obesity (BMI and WC), which are easily applied. The results showed a significant association of the anthropometric parameters (BMI and WC) with physical inactivity, which has been observed in other studies,^6^^,^ 15 ^-^ 17 showing that children/adolescents with excess weight are less active when compared to those with normal weight.

Considering this picture, two questions arise: Are the students overweight and obese because they are less active? Or are they less active due to overweight/obesity? Literature has yet to elucidate this issue, as obesity is a multifactorial trait and may be related to other aspects such as sleep, diet and endogenous factors. However, it is known that adequate levels of physical activity not only prevent obesity, but also the associated metabolic diseases, being a crucial variable in the prevention and control of body weight.^18^^-^ 22

Studies carried out in Brazil have shown a prevalence of physical inactivity in 10-94% of young individuals in different age ranges, using different research tools,^14^^,^ 23 ^-^ 26 making it impossible to compare results of the physical activity level, but pointing to the urgent need for public health strategies to reduce its impact as a cardiovascular disease risk factor and cause of death.

The association between physical activity level and the two anthropometric measures used in this study does not rule out their importance as potential predictors of physical inactivity. However, the strength of the results may have been affected, as the analysis of the physical activity level was not performed with an accelerometer, which is currently the most reliable method to estimate the level of physical activity.^27^ In the present study, the analysis was performed indirectly, i.e., through a questionnaire, in which students reported the time of weekly physical activity. It is noteworthy the fact that the questionnaire was applied once in each student, and the students may have overestimated or underestimated their actual time of physical activity per week, thus characterizing a possible classification bias and a limitation of this study.

Another factor that might explain the existing conflicts in the literature about the best anthropometric indicator in the association with physical inactivity is the strong association found between the two indicators (BMI and WC) in children and adolescents.^28^ The high rates of BMI may be related to excess body fat, due to the fact that the proportion of lean mass is not very significant in this age group.

We also emphasize the analysis of a possible association between the two age categories (10-12 and 13-14 years) and physical inactivity in the students analyzed in the present study. However, the results showed no significant association (*p*=0.058), unlike some studies that have found a trend of physical inactivity in older ages, even with little difference between these age groups.^6^^,^ 29 To further elucidate this question, the analysis of sexual maturation would be necessary; however, due to bureaucratic issues in some schools, it was not possible to carry out such an analysis, which is another limiting factor in this study.

Regarding gender, this study showed no significant difference between the groups, once again contrary to previous studies, in which females showed significantly higher proportion of physical inactivity.^6^^,^ 14 ^,^ 25 One characteristic found in five of the six schools was the separation of girls and boys during Physical Education classes, which may have somehow influenced the results of this variable. According to the report of the teachers, after schools started separating the genders during classes, the girls began to participate more effectively in practical classes and possibly joined the practice of physical activity outside the school.

Still on the school environment, we analyzed the type of school, either private or public, and physical inactivity. The results found no association between these variables, which has been already demonstrated in the study involving schoolchildren in the city of Maceió, state of Alagoas, Brazil.^29^ In all analyzed schools, students had physical education classes once a week, with two consecutive 50-minute classes (100 min). Considering that around 30% of the whole Physical Education class corresponds to physical activities of moderate/vigorous intensity,^30^ students would need 270 minutes of physical activity outside the school environment to be considered physically active. This time, divided into the six days of the week when students do not have P.E. classes, would total 45 minutes of moderate/vigorous physical activity per day.

In conclusion, the results showed a significant association of physical inactivity with the analyzed anthropometric measures (BMI and WC), implying that inadequate levels of physical activity seem to be a factor triggered by excess weight and abdominal adiposity or vice-versa. One can also verify that there was no significant association of physical inactivity with gender and age, which is an important finding in this study. 

The lack of association of these variables can help in the organization and implementation of future interventions in students that had unsatisfactory levels of physical activity. In this sense, preventive measures need to be part of the public health policies and programs, especially in the school environment, emphasizing the importance of physical exercise in the control and reduction of weight and body composition. 

Further studies need to be carried out with more reliable methods (accelerometers), with students of different levels of education (preschool to College/University) in an attempt to establish a profile of the physical activity level at each stage of education. Another suggestion is carrying out an analysis of the separation of physical education classes between boys and girls and their association with adherence to physical activity within the school environment as well as outside.
