# Supplementary material for: Physical inactivity and anthropometric measures in schoolchildren from Paranavaí, Paraná, Brazil
Source: Rev Paul Pediatr. 2015 Mar;33(1):50–5. doi: 10.1016/j.rpped.2014.11.009 (PMC4436956; doi:10.1016/j.rpped.2014.11.009)
Supplement: Translation

# Translation: Inatividade física e medidas antropométricas em escolares de Paranavaí, Paraná, Brasil

**Authors:** Flávio Ricardo Guilherme, Carlos Alexandre Molena-Fernandes, Vânia Renata Guilherme, Maria Teresa Martins Fávero, Eliane Josefa Barbosa dos Reis, Wilson Rinaldi

**Affiliations:** a Universidade Estadual de Maringá (UEM), Maringá, PR, Brasil; b Universidade Estadual do Paraná (Unespar/Fafipa), Paranavaí, PR, Brasil

**Keywords:** Estilo de vida sedentário, Obesidade, Criança, Adolescente

## Abstract

**OBJETIVO:**

Investigar a associação entre a inatividade física e medidas antropométricas em escolares de Paranavaí, Paraná, Brasil.

**MÉTODOS:**

Pesquisa com delineamento transversal, feita em julho e agosto de 2013. Amostra composta por 566 escolares (287 meninos e 278 meninas), de 10 a 14 anos, do 6° ao 9° ano da rede pública e privada de Paranavaí (PR). As variáveis analisadas foram: tempo de atividade física semanal, por meio de questionário (inatividade física: < 300 min/semanal), índice de massa corporal (IMC) e circunferência de cintura (CC). Na análise estatística foram usados os testes U de Mann-Whitney e *t* de Student para comparar os sexos. Para verificar os fatores associados ao nível insuficiente de atividade física aplicou-se o modelo de regressão logística binária univariada e multivariada, expressa em *odds ratio* (OR), e intervalo de confiança de 95% (IC95%).

**RESULTADOS:**

Houve associação entre inatividade física e as medidas antropométricas para IMC (*p*<0,001) e CC (*p*<0,001), com prevalências de 56,1% e 52,7% de inativos, respectivamente. Na análise multivariada, foram observadas associações significativas de inatividade física nos alunos que apresentaram excesso de peso (OR 1,8; IC95%: 1,1-3,0) e circunferência de cintura aumentada (OR 2,2; IC95%: 1,4-3,8).

**CONCLUSÕES:**

Nível inadequado de atividade física é fator determinante no excesso de peso e na adiposidade abdominal. Nesse sentido, medidas preventivas devem ser tomadas, principalmente nas escolas, e enfatizar-se a importância do exercício físico no controle da composição corporal e redução do pesoe.

## Introdução

A obesidade pode ser definida, de forma simplificada, como uma doença caracterizada pelo acúmulo excessivo de gordura corporal, consequência do balanço energético positivo.^1^ Nas últimas décadas, sua prevalência aumentou em todo mundo e tornou um grande problema de saúde.^2^

Dados recentes têm demonstrado um acréscimo substancial nos casos de sobrepeso e obesidade durantes os últimos 20 anos já na infância e adolescência.^3^ Uma possível explicação seria que, nessa fase da vida, ocorrem mudanças biológicas e comportamentais, entre elas a adoção de hábitos alimentares inadequados, como o aumento do fornecimento de energia pela dieta e a inatividade física.^4^ ^and^ 5

No que se refere à inatividade física, o Brasil apresenta um quadro preocupante na população jovem, devido às altas prevalências encontradas.^6^ ^and^ 7 Essa preocupação é ainda agravada por evidências de que o nível de atividade física tende a diminuir com o passar da idade, o que é conhecido como *tracking* da atividade física. 6

Pequenos aumentos na atividade física estão associados com benefícios à saúde. Em crianças e adolescentes, a atividade física pode reduzir os sintomas de depressão e estresse, melhorar a função cardiorrespiratória, a aptidão muscular e a saúde óssea e diminuir os níveis de gordura corporal,^8^ esse o principal fator de risco para o surgimento de doenças metabólicas.

Como a inatividade física é habitualmente adquirida na infância e permanece na vida adulta, é fundamental a sua identificação precoce e associação com indicadores antropométricos para um melhor controle e prevenção do excesso de peso e suas comorbidades na vida adulta. Portanto, este estudo teve como objetivo investigar a associação entre a inatividade física e medidas antropométricas em escolares de Paranavaí-Paraná, Brasil.

## Método

A população foi composta por estudantes da rede pública e privada do Ensino Fundamental II da cidade de Paranavaí (PR). De acordo com dados do Núcleo Regional de Educação de Paranavaí, em 2013 4.540 alunos estavam matriculados em oito escolas públicas e quatro privadas da cidade. Paranavaí fica na Região Noroeste do Estado do Paraná. Em 2010, o município tinha 81.590 habitantes, dos quais 95,3% residiam na zona urbana. O desempenho municipal classificava-se como médio para os quesitos emprego, renda, produção agropecuária e saúde e alto para o quesito educação. O IDH atual é de 0,763 e o PIB per capita de R$14.180,10.

Esta pesquisa com delineamento transversal foi feita em julho e agosto de 2013. A amostra foi composta da população escolar de 6° ao 9° ano, de 10 a 14 anos, proveniente de escolas da rede pública (quatro) e privada (duas) do município. Tais escolas corresponderam a 50% do total de escolas e representaram 61% da população escolar do município. As turmas foram escolhidas por amostragem aleatória sistemática, em três etapas: 1) sorteio de uma escola de cada região da cidade para melhor caracterizar o universo escolar da cidade, tendo em vista que, em todas as regiões, o número de escolas e alunos foi semelhante; 2) sorteio das turmas em cada escola; 3) convite a todos os escolares das turmas sorteadas e explicações sobre o estudo.

O cálculo amostral resultou do número do total da população analisada (n=4540); prevalência do desfecho (inatividade física) de 50% (desconhecida); nível de confiança igual a 95%; e erro amostral de 5%. Com base nesses parâmetros, estimou-se coletar dados de 354 escolares. Foram acrescentados na amostragem 10%, com previsão de eventuais perdas e recusas, e 10% para as análises multivariadas, o que resultou 425 crianças e adolescentes. As avaliações foram feitas somente naqueles escolares convidados, que aceitaram participar da pesquisa e que apresentaram o Termo de Consentimento Livre e Esclarecido assinado pelos responsáveis, 578 alunos. Desses, excluíram-se 12: 1) idade diferente de 10 a 14 anos; 2) não feitura de todas as avaliações. A amostra final foi composta por 566 crianças e adolescentes, 287 meninos e 279 meninas. A margem de erro amostral calculada *a posteriori* foi de 3,8 a 3,9%, abaixo do valor estabelecido *a priori* (5%).

As avaliações foram feitas durante o horário escolar, por avaliadores treinados e com equipamentos calibrados. A estatura foi medida com estadiômetro de parede (Wisoâ, Brasil) com resolução de 0,1 cm e a massa corporal em balança digital (G-Tech) com capacidade máxima de 150 kg e resolução de 100 g. O avaliado vestiu somente o uniforme escolar, sem casaco ou objetos nos bolsos. O IMC (kg/m2) foi usado para classificar os escolares com peso adequado e excesso de peso.^9^ Os dados dos escolares com baixo peso (0,3%; n = 1) foram incluídos na categoria de peso adequado.

A circunferência de cintura foi obtida com o uso de fita métrica flexível e inextensível (Gulick, Brasil), com resolução de 0,1 cm, aplicada imediatamente acima das cristas ilíacas. Para a classificação de obesidade abdominal (central), usou-se o ponto de corte de *p*≥75 para todas as etnias. 10 Vale ressaltar que poucos estudos têm usado parâmetros nacionais para classificar obesidade abdominal em adolescentes. Para o nível de atividade física, foi usado o questionário aplicado pelo Instituto Brasileiro de Geografia e Estatística (IBGE) na Pesquisa Nacional de Saúde do Escolar*.* 11 A inatividade física foi considerada conforme o ponto de corte de < 300 minutos de atividade física moderada/vigorosa semanal de acordo com a diretriz de atividade física para adolescentes. 12

Na análise estatística, foi usado o teste de teste de Kolmogorov-Smirnov para identificar a normalidade dos dados. Para comparar as características antropométricas e o tempo de atividade física semanal entre os sexos, usou-se o teste *U* de Mann-Whitney para amostras independentes não paramétricas e o teste *t* de Student para amostras independentes paramétricas, acompanhado do teste de Lèvene para análise da homogeneidade das variâncias.

O teste do qui-quadrado foi adotado para verificar diferenças na proporção de inatividade física (variável dependente), segundo as categorias das variáveis independentes. A análise exploratória dos dados demonstrou relação não linear entre *x* e *y*, a partir de determinado ponto de sua distribuição, assumindo curva logística em S. Efetuou-se, portanto, a regressão logística binária univariada e multivariada, determinou-se a razão de chances ou *odds ratio* (OR) e os respectivos intervalos de confiança (95%), no intuito de analisar a associação de inatividade física (variável dependente) com as variáveis independentes*.* Todas as variáveis estudadas foram dicotomizadas e o critério para inclusão das variáveis independentes no modelo multivariado foi um nível de associação de *p*≤0,20 com a variável dependente, pelo teste qui-quadrado.

As análises foram feitas por meio do Statistical Package for a Social Science (SPSS), versão 20.0, considerando-se p < 0,05. Este estudo foi aprovado pelo Comitê de Ética em Pesquisa da Universidade Estadual de Maringá, sob parecer número 353.552, consoante a Declaração de Helsinki.

## Resultados

Dos 566 alunos selecionados para a pesquisa, 50,7% (287) eram do sexo masculino e 49,3% (279) do feminino, com médias (± desvio padrão) para idade de 12,4 (±1,2) e 12,3 (±1,2) anos, massa corporal de 52 (±13,8) e 49,5 (±11,7) kg, estatura 1,59 (±0,1) e 1,56 (±0,1) cm, IMC de 20,29 (±3,8) e 20,19 (±4,2), circunferência de cintura 74,4 (±11,2) e 71,6 (±10,7) cm e, por fim, tempo de atividade de 359 (±401,4) e 343 (±471,2) respectivamente. Os meninos demonstraram médias significativamnete superiores de massa corporal, estatura e circunferência de cintura em relação às meninas (*p*≤0,05). As médias de idade, o IMC e o tempo de atividade física foram semelhantes entre os sexos (tabela 1).

**Tabela 1:** Idade, características antropométricas e de atividade física nos escolares de Paranavaí, Paraná em 2013

Variáveis	Média ± DP		*p* valor
	Masculino (n=287)	Feminino (n=278)	
Idade (anos)	12,4±1,2	12,3±1,2	0,256
Massa (kg)	52,0±13,8	49,5±11,7	0,048
Estatura (cm)	1,59±0,1	1,56±0,1	0,002
IMC (kg/m2)	20,29±3,8	20,19±4,2	0,788
CC (cm)	74,4±11,2	71,6±10,7	0,002
Tempo AF (minuto/semana)	359±401,4	343±471,2	0,170

AF, atividade física.

Na tabela 2, pode-se observar que a maioria dos inativos fisicamente era do sexo masculino (53,3%), com idade entre 10 e 12 anos (59,4%) e da rede pública de ensino (67,8%). No entanto, as únicas variáveis independentes associadas com níveis inadequados de atividade física (< 300 min/semana) foram o IMC (*p≤*0,001) e a CC (*p≤*0,001), com prevalências de 56,1% e 52,7% de inativos, respectivamente.

**Tabela 2:** Características sociodemográficas e antropométricas em relação ao nível de atividade física nos escolares de Paranavaí, Paraná em 2013 (n=566)

Variáveis	n	Nível de atividade física		
		Total (%)	Insuficiente Ativos (%)	*p* valor
*Sexo*				
Feminino	279	49,3	46,9	0,323
Masculino	287	50,7	53,1	

*Idade*				
10-12 anos	310	54,8	59,4	0,058
13-14 anos	256	45,2	40,6	

*Rede*				
Pública	381	67,3	67,8	0,839
Privada	185	32,7	32,4	

*IMC*				
Eutróficos	339	59,9	43,9	0,001
Excesso de Peso	227	40,1	56,1	

*CC*				
Adequado	361	63,8	47,3	0,001
Obesidade	205	36,2	52,7	

IMC, índice de massa corporal; CC, circunferência de cintura.

Em seguida, na análise univariada, pôde-se observar que a inatividade física apresentou-se positivamente associada com o IMC (OR 3,2; IC95%: 2,3-4,6) e CC (OR 3,5; IC95%: 2,5-5,0).

Na análise multivariada, por meio de regressão logística, verificou-se que o modelo com maior validade preditiva incluiu as variáveis IMC, CC e idade (índice de ajuste do modelo de Hosmer e Lemeshow = 0,938), com capacidade de explicar 79,5% dos casos de alunos com nível de atividade física adequada. A inatividade física insuficiente foi positivamente associada novamente com as duas variáveis antropométricas ajustadas pela idade. Alunos classificados como portadores de IMC aumentado (excesso de peso) tiveram 1,8 vez (IC 95%: 1,8-3,0) mais chance de ser inativos fisicamente em relação aos eutróficos. Quanto à CC, alunos com obesidade abdominal tiveram 2,2 vezes (IC 95%: 1,4-3,8) mais chance de apresentarem níveis inadequados de atividade física. (tabela 3).

**Tabela 3:** Fatores associados ao nível insuficiente de atividade física nos escolares de Paranavaí, medido por meio de regressão logística

Variáveis	*Odd ratio* bruto (IC 95%)	*Odd ratio* ajustado (IC 95%)
*Sexo*		
Masculino	1	-
Feminino	0,9 (0,6-1,2)	-

*Idade*		
10-12 anos	1	1
13-14 anos	0,7 (0,5-1,0)	0,9 (0,6-1,3)

*Rede*		
Pública	1	-
Privada	1 (0,7-1,4)	-

*IMC*		
Eutróficos	1	1
Excesso de Peso	3,2 (2,3-4,6)	1,8 (1,1-3,0)

*CC*		
Adequado	1	1
Obesidade	3,5 (2,5-5,0)	2,2 (1,4-3,8)

IMC, índice de massa corporal; CC, circunferência de cintura.

## Discussão

A inatividade física na população infantojuvenil tem aumentado ao redor do mundo, são observadas maiores proporções nos indivíduos com excesso de peso.^6^ ^and^ 8 Estudos tentaram identificar o parâmetro antropométrico de melhor associação com níveis de atividade física em crianças e adolescentes, mas os resultados são conflitantes.^13^ ^and^ 14 Esta pesquisa buscou evidenciar essa questão, analisar a relação entre a inatividade física com dois indicadores antropométricos de obesidade (IMC e CC) de fácil aplicação. Os resultados mostraram associação significativa dos parâmetros antropométricos (IMC e CC) com a inatividade física, o que já foi observado em outros trabalhos,^6^^,^ 15 ^,^ 16 ^and^ 17 o que mostra que as crianças/adolescentes com excesso de peso são menos ativos em relação aos eutróficos.

Diante desse quadro, surgem duas perguntas: "Será que os alunos estão com excesso de peso e obesidade por serem menos ativos?" "Ou são menos ativos devido ao excesso de peso/obesidade?" Na literatura ainda não foi elucidada essa questão, pelo fato de a obesidade ser multifatorial, pode estar relacionada com outros aspectos, como sono, dieta e fatores endógenos. No entanto, sabe-se que níveis adequados de atividade física previnem não somente a obesidade, como também as doenças metabólicas relacionadas, essa é uma variável determinante na prevenção e no controle do peso corporal.^18^^,^ 19 ^,^ 20 ^,^ 21 ^and^ 22

Trabalhos feitos no Brasil demonstraram uma prevalência de sedentarismo entre 10-94% em jovens de diversas faixas etárias e com diferentes instrumentos de investigação,^14^^,^ 23 ^,^ 24 ^,^ 25 ^and^ 26 o que impossibilita a comparação de resultados do nível de atividade física, mas aponta para a urgente necessidade de estratégias de saúde pública para a redução do seu impacto como fator de risco de doenças cardiovasculares e causas de morte.

A associação entre o nível de atividade física e as duas medidas antropométricas evidenciadas neste estudo não descarta sua importância como potencias preditores de inatividade física. No entanto, a força dos resultados pode ter sido afetada devido à análise do nível de atividade física não ter sido feita com acelerômetros, esse o método atualmente mais fidedigno para estimar o nível de atividade física.^27^ Na presente pesquisa, a análise foi feita de maneira indireta, ou seja, por meio da aplicação de um questionário no qual os alunos relatavam o tempo de atividade física semanal. Vale ressaltar que o questionário foi aplicado uma vez em cada aluno, pôde o aluno superestimar ou subestimar seu real tempo de atividade física semanal, o que caracteriza um possível viés de classificação e uma limitação deste estudo.

Outro fator que pode explicar os conflitos existentes na literatura acerca do melhor indicador antropométrico na associação com inatividade física é a forte relação encontrada entre os dois indicadores (IMC e CC) em crianças e adolescentes.^28^ Os elevados índices de IMC podem estar relacionados com excesso de gordura corporal devido ao fato de a proporção de massa magra não ser muito expressiva nessa faixa etária.

Ressalta-se ainda a análise feita para uma possível associação entre duas categoria de idade (10-12 e 13-14 anos) e inatividade física nos estudantes aqui analisados. No entanto, os resultados não apresentaram associação significativa (*p*=0,058), contradizem alguns estudos que encontraram tendência de inatividade física em idade maiores, mesmo com pouca diferença entre essas faixas etárias. 6 ^and^ 29 Para melhor elucidar essa questão, a análise da maturação sexual seria necessária; porém, devido a questões burocráticas de algumas escolas, não foi possível fazer tal análise, é esse outro fator limitante nesta pesquisa.

Em relação ao gênero, a presente pesquisa não apresentou diferença significativa entre os grupos, novamente em contradição com os achados da literatura, em que o sexo feminino apresenta proporções de inatividade física significativamente maiores.^6^^,^ 14 ^and^ 25 Uma característica encontrada em cinco das seis escolas foi a divisão de meninas e meninos nas aulas de educação física, o que pode, de alguma maneir, ter influenciado os resultados dessa variável. Segundo relato das professoras, após as escolas adotarem essa divisão, as meninas começaram a participar de maneira mais efetiva nas aulas e possivelmente aderiram à prática de atividade física fora da escola.

Ainda sobre o ambiente escolar, analisou-se o tipo de escola, privada ou pública, e a inatividade física. Os resultados não encontraram associação entre essas variáveis, o que já foi evidenciado no estudo que envolveu escolares em Maceió.^29^ Em todas as escolas analisadas, os alunos tinham aulas de educação física uma vez por semana, duas aulas de 50 minutos seguidas (100 minutos). Ao considerar que em torno de 30% do total da aula de educação física corresponde a atividades físicas de moderada/vigorosa intensidade,^30^ os alunos precisariam de 270 minutos de atividade física fora do ambiente escolar para ser considerados ativos fisicamente. Esse tempo, dividido nos seis dias que os alunos não têm aula, totalizaria 45 minutos de atividade física moderada/vigorosa por dia.

Em conclusão, os resultados mostraram associação significativa de inatividade física com as medidas antropométricas analisadas (IMC e CC), inferindo que níveis inadequados de atividade física parecem ser um fator desencadeado pelo excesso de peso e adiposidade abdominal ou vice-versa. Pôde-se verificar, ainda, que não houve associação significativa de inatividade físca com o gênero e a idade, esse um importante achado no estudo. A não associação dessas variáveis pode ajudar na organização e aplicação de futuras intervenções nos alunos que apresentaram níveis insatisfatórios de atividade física. Nesse sentido, medidas preventivas precisam fazer parte das políticas e programas de saúde pública, principalmente no ambiente escolar, e enfatizar a importância do exercício físico no controle e na redução do peso e composição corporal.

Novos estudos precisam ser feitos com métodos mais fidedignos (acelerômetros), com estudantes de diferentes graus de escolaridade (pré-escola ao ensino superior), na tentativa de traçar um perfil de nível de atividade física em cada estágio de ensino. Outra sugestão é uma análise da divisão das aulas de educação física entre meninos e meninas e sua relação com a adesão à prática de atividade física no ambiente escolar e fora dele.
